# Altered Sympathetic-to-Immune Cell Signaling via ****β****_**2**_-Adrenergic Receptors in Adjuvant Arthritis

**DOI:** 10.1155/2013/764395

**Published:** 2013-10-01

**Authors:** Dianne Lorton, Denise L. Bellinger, Jill A. Schaller, Eric Shewmaker, Tracy Osredkar, Cheri Lubahn

**Affiliations:** ^1^Hoover Arthritis Research Center, Banner Health Research Institute, 10515 W. Santa Fe Drive, Sun City, AZ 85351, USA; ^2^Department of Psychology, Kent State University, 133 Kent Hall, Kent, OH 44242, USA; ^3^Kent-Summa Institute for Clinical and Translational Research, Summa Health System, 525 East Market Street, Akron,OH 44304, USA; ^4^Department of Pathology and Human Anatomy, Alumni Hall for Basic Sciences, Loma Linda University School of Medicine, 11021 Campus Street, Loma Linda, CA 92354, USA

## Abstract

Adjuvant-induced arthritic (AA) differentially affects norepinephrine concentrations in immune organs, and *in vivo* **β**-adrenergic receptor (**β**-AR) agonist treatment distinctly regulates *ex vivo* cytokine profiles in different immune organs. We examined the contribution of altered **β**-AR functioning in AA to understand these disparate findings. Twenty-one or 28 days after disease induction, we examined **β**_2_-AR expression in spleen and draining lymph nodes (DLNs) for the arthritic limbs using radioligand binding and western blots and splenocyte **β**-AR-stimulated cAMP production using enzyme-linked immunoassay (EIA). During severe disease, **β**-AR agonists failed to induce splenocyte cAMP production, and **β**-AR affinity and density declined, indicating receptor desensitization and downregulation. Splenocyte **β**_2_-AR phosphorylation (p**β**_2_-AR) by protein kinase A (p**β**_2_-AR_PKA_) decreased in severe disease, and p**β**_2_-AR by G protein-coupled receptor kinases (p**β**_2_-AR_GRK_) increased in chronic disease. Conversely, in DLN cells, p**β**_2_-AR_PKA_ rose during severe disease, but fell during chronic disease, and p**β**_2_-AR_GRK_ increased during both disease stages. A similar p**β**_2_-AR pattern in DLN cells with the mycobacterial cell wall component of complete Freund's adjuvant suggests that pattern recognition receptors (i.e., toll-like receptors) are important for DLN p**β**_2_-AR patterns. Collectively, our findings indicate lymphoid organ- and disease stage-specific sympathetic dysregulation, possibly explaining immune compartment-specific differences in **β**_2_-AR-mediated regulation of cytokine production in AA and rheumatoid arthritis.

## 1. Introduction 

The sympathetic nervous system (SNS) via its modulation of the immune system is an important player in determining the disease onset, progression, and severity in rheumatoid arthritis (RA) and animal models of RA [1, 2]. Autonomic dysregulation is reported in RA, juvenile chronic arthritis, and arthritic animals (reviewed in [3]) that support increased sympathetic activity. Treatment of rodent RA models with adrenergic drugs suggests both a permissive and suppressive role for the SNS in the pathogenesis of RA [4–6]. Blocking sympathetic signaling at disease initiation with an arthrotigenic challenge or disease onset exacerbates or attenuates disease severity [5, 7], respectively, suggesting a complex role for the SNS in RA. 

SNS dysregulation in RA patients is supported by reports of reduced numbers of *β*_2_-AR receptors and functional responses of peripheral blood mononuclear cells (PBMCs) to sympathomimetic agents compared with nonarthritic, age-matched controls [8–12]. The anti-CD3 antibody, OKT3-induced PBMC proliferative responses in the presence of catecholamines are attenuated in RA compared with healthy subjects [8]. Diminished expression and activity of G protein-coupled receptor kinases (GRKs) in PBMCs occur in RA patients [13] and in animal models of RA [14]. In RA patients, PBMC GRK-2 and GRK-6 levels are reduced, whereas, GRK-5 levels are not altered. Given that receptor ligand concentration and GRKs regulate *β*-AR functions, these findings indicate that sympathetic signaling in targeted immune cells is dysregulated in RA and animal models of RA. 

Sympathetic nerves signal immune cells in lymphoid organs primarily through the release of norepinephrine (NE). The primary adrenergic receptors (ARs) expressed on lymphocytes are *β*_2_-ARs. *β*-ARs are G coupled-protein receptors that classically use the cAMP/protein kinase A (PKA) signaling pathway. The expression of *β*_2_-AR on target cells is dynamically regulated, in part by ligand availability, but also intracellularly by PKA, *β*-arrestins, and different GRKs [15]. 

*β*_2_-AR function is regulated by PKA and GRK through *β*_2_-AR phosphorylation (p*β*_2_-AR) at different serines [16–18]. Desensitization is induced by PKA, and at higher agonist concentrations, by PKA and GRK [19–21]. PKA-induced p*β*_2_-AR (p*β*_2_-AR_PKA_) alters receptor conformation, impairing Gs and enhancing Gi protein coupling to induce transient activation of extracellular signal-regulated kinases (ERK 1/2) [21]. p*β*_2_-AR by GRK (p*β*_2_-AR_GRK_), specifically GRK2, leads to *β*-arrestin binding to the *β*_2_-AR [21–23], altering the conformation of the *β*_2_-AR, further impeding Gs protein coupling [21] and inducing receptor internalization. Internalized *β*_2_-AR are dephosphorylated and recycled to the plasma membrane or degraded [24, 25]. 

Additionally, *β*_2_-AR signaling can induce sustained ERK 1/2 signaling in a *β*-arrestin- [15, 20, 26] and GRK-dependent manner [27, 28]. p*β*_2_-AR_GRK_ occurs in an agonist concentration-dependent manner by GRK 2, 5, and 6 to induce different receptor functions [18, 28, 29]. High agonist concentrations induce PKA- and GRK5/6-mediated p*β*_2_-AR and subsequent binding to *β*-arrestin [30], a scaffold/adaptor protein for mitogen-activated protein kinase (MAPK) activation [31–33]. In this manner, high agonist concentrations can induce sustained ERK activation independent of the G protein pathway [27, 34, 35]. Therefore, chronically elevated sympathetic tone in patients with RA [36–39] may provide conditions for differential p*β*_2_-AR that result in *β*_2_-AR-induced ERK 1/2 signaling. 

Previously, our laboratory has reported reduced and elevated NE concentration in the spleen and draining lymph nodes (DLN) of adjuvant-induced arthritis (AA) rats, respectively, compared with non-AA controls [40]. These findings suggest differential activational states of splenic and DLN sympathetic nerves in AA, which may induce differential *β*_2_-AR signaling in immune cells from these two lymphoid organs. Consistent with this hypothesis, in rats challenged to induce AA, a model of RA, we find that TH1 versus TH2 cytokine production differs depending on the source of immune cells cultured *ex vivo* [41]. Despite findings that support the downregulation and desensitization of *β*_2_-ARs in peripheral blood leukocytes from RA patients, no studies have examined the extent to which leukocyte *β*_2_-ARs in animal models of RA mimic the receptor changes observed in RA. It is also not clear if leukocytes from relevant lymphoid organs, where autoreactive T cells differentiate, also demonstrate changes in *β*_2_-AR indicative of altered signaling and how such changes impact immune functions in different lymphoid organs in RA or animal models of this disease. In the present study, we tested the hypothesis that disparate TH cell cytokine profiles in different lymphoid organs reflect disease-induced and organ-specific changes of *β*_2_-AR functions. We examined *β*_2_-AR numbers, affinity, signaling, and phosphorylation patterns in immune cells from spleens and DLN of AA rats challenged with complete Freund's adjuvant (CFA) and non-AA rats challenged with components of the CFA (mineral oil or bacterial cell wall) or saline vehicle. We report here immune organ-dependent changes in *β*_2_-AR expression, intracellular signaling, and receptor phosphorylation in AA rats and nonarthritic rats treated with one of the components of CFA, mineral or the heat-killed bacterial cell wall in saline. 

## 2. Materials and Methods

### 2.1. Materials

Dried and heat-killed *Mycobacterium butyricum* (*M. butyricum*) and mineral oil were obtained from Difco (Detroit, MI) and Sigma-Aldrich Company (St. Louis, MO), respectively. CFA and *M. butyricum* in saline were prepared as previously described [5]. All tissue culture media and supplements were obtained from Gibco BRL (Rockville, MD), unless otherwise stated. EIA kits for cAMP determination and (^125^I)CYP (2200 Ci/mMole) were purchased from R&D Systems and Amersham International (Amersham Bucks, UK), respectively. OptEIA kits for the detection of interferon-*γ* (IFN-*γ*) were obtained from BD Pharmingen (Los Angeles, CA). Antibodies recognizing the *β*_2_-AR (no. sc-569) and p*β*_2_-AR at serines 345 or 346 (PKA site; no. 16718) or 355 or 356 (GRK site; no. 16719) were purchased from Santa Cruz Biotechnology (Santa Cruz, CA). CGP-12177, propranolol, and all chemicals, reagents, and buffers used for the western blotting were purchased from Sigma-Aldrich (St. Louis, MO) unless stated otherwise. 

### 2.2. Animals

Male Lewis rats (200–250 g) were obtained from Charles River Laboratory (Wilmington, MA). Rats were housed two per cage in plastic bottom cages with soft CareFRESH bedding (Absorption, Bellingham, WA), allowed access to water and food pellets *ad libitum*, and placed on a 12 h-on 12 h-off light schedule. Rats were allowed to adjust to conditions in the Banner Sun Health Research Institute's AAALAC accredited vivarium for one week prior to starting the experiments. For AA rats, food (Purina Lab Diet 5001) was placed in the bottom of the cage, and water was supplied using long-stemmed sipper tubes to ensure easy access to food and water. All rats were observed to eat and drink throughout the study. Animals were weighed weekly and observed daily to verify adequate weight gain and overall good health. Other than the development of arthritis, the general health of the animals was maintained during the course of the experiments. Protocols for the use and care of animals in this study were approved prior to beginning the experiments by the Banner Health Research Institute's Institutional Animal Care and Use Committee and complied with NIH guidelines for the humane use and care of research animals.

### 2.3. Induction of AA and Experimental Design

Rats were randomly assigned to 1 of 4 groups (*n* = 4 per group per sacrifice day) based on the treatment they received. The experiment was repeated twice per time point. No differences were seen between the experiments so the data for the two experiments was collapsed giving an *n* = 8 per group, per time point. Rats were given 0.1 mL (1) complete Freund's adjuvant (CFA); (2) mineral oil (MO); (3) *M. butyricum* (SMB; 30 mg/10 mL sterile endotoxin-free saline); or (4) sterile endotoxin-free saline by intradermal injection into the base of the tail. Only rats that received CFA developed AA. Control rats treated with MO or SMB served to control for the CFA vehicle or the effects of mycobacterium challenge in the absence of inflammatory arthritis, respectively. Saline-treated rats controlled for the stress of the injection and handling. A single preparation of CFA and SMB was used for this study. All CFA-immunized animals developed arthritis with similar timing of disease onset and severity. After overdose-induced anesthesia with 8% chloral hydrate, rats were sacrificed on day (D) 21 or 28 after CFA challenge or control treatments. The DLN (popliteal and inguinal lymph nodes) and spleens were harvested, and single cell suspensions were prepared. D21 and D28 represent acute and severe disease, respectively.

A separate experiment was performed to evaluate whether altered *β*_2_-AR phosphorylation in immune organs from AA rats affects the production of a cytokine that is regulated by *β*_2_-AR under normal conditions and in AA. The effects of *in vivo* treatment of arthritic rats with a *β*_2_-AR agonist, terbutaline, or vehicle on *ex vivo* production of IFN-*γ* by spleen and DLN cells were examined. Terbutaline (1200 *μ*g/day) was administered by intraperitoneal (i.p.) injections twice a day in a total volume of 250 *μ*L per injection (*n* = 8). Control arthritic rats were given i.p injections of the same volume of vehicle (0.01 mM ascorbic acid in 0.9% sterile, endotoxin-free saline; *n* = 8). Terbutaline or vehicle treatments were started on day 12 after immunization, the time of disease onset, and continued until sacrifice. The animals were sacrificed using an overdose of 8% chloral hydrate 10.0 mL/kg body weight. On day 28, DLN and spleen were dissected and collected in preparation for culturing immune cells. 

### 2.4. Disease Assessment

The onset and progression of arthritis was apparent based on the development of inflammation and swelling of the hind and fore limbs. The inflammatory response in arthritic rats was assessed by routine methods, as previously described [42]. Dorsoplantar widths of the hind feet were measured using a dial thickness gauge (Mitutoyo Corporation, Chicago, IL), beginning before CFA immunization and continuing approximately every other day until sacrifice. After sacrifice, the hind limbs were removed, and radiographs were taken to assess disease severity using the following settings: 400 nN, 50 kvp, and 0.4 s exposure time at 40 cm. The digitized images are printed using a Fujifilm model FM-DPL printer for analysis. 

X-rays were evaluated using a grading scale, as previously reported [5]. In brief, the radiographs were coded to obscure the treatment groups, and then two independent observers subjectively rated each of the radiographs using the scale: 0 (normal), 1 (slight), 2 (mild), 3 (moderate), and 4 (severe) abnormalities in the tissue. The radiographs were scored using the 4 point scale for each of the following characteristics: (i) swelling as indicated by the width of soft tissue shadows and changes in the normal configuration of the soft tissue planes; (ii) osteoporosis as measured by bone density (recognized by increases in radiolucency relative to uninvolved adjacent bone); (iii) cartilage loss shown by narrowing of the joint spaces; (iv) heterotopic ossification defined as proliferation of new bone tissue (fine ossified line paralleling normal bone but not contiguous with calcified area of the bone itself); and (v) bone erosions. The radiographic scores for each category were added for both hind limbs giving a maximum score of 40. 

### 2.5. Harvesting Spleen and Lymph Node Cells

After anesthetic overdose, the spleens and DLN were aseptically removed. Spleens were placed into stomacher bags containing 10 mL Hank's balanced salt solution (HBSS) and homogenized for 2 × 30 sec. Spleen cells were triturated with a 10 mL pipette, then washed with an additional 10 mL of HBSS, and passed through a 70 *μ*m nylon mesh (Marsh Industries, Plano, TX) to remove the extraneous connective tissue. The collected cells were centrifuged at 3,000 g for 7 min and then resuspended in 5 mL of a NH_4_Cl hypotonic buffer for 3 min to lyse the red blood cells. The immune cells were washed 2X with 10 mL HBSS, centrifuged, and resuspended into complete media (RPMI 1640 supplemented with 5% fetal calf serum and 1% antibiotic/antimycotic). Splenocytes were counted using a hemocytometer and then brought to a final concentration of 2 × 10^6^ cells/mL in complete media. Single cell lymph node suspensions were prepared by teasing apart the harvested lymph nodes in 5 mL HBSS using fine forceps. These cells were then triturated with a 5 mL pipette to disperse immune cells and washed with an additional 5 mL HBSS. Cells and tissue debris were separated by filtering the cell suspension through a 70 *μ*m nylon mesh. The cells were pelleted and resuspended in 5 mL HBSS. The cells were pelleted and resuspended into complete media, counted using a hemocytometer, and brought to a final concentration of 2 × 10^6^ cells/mL.

### 2.6. Measurement of cAMP by Enzyme Immunoassay (EIA)

To measure cAMP production, spleen cells were incubated in the presence or absence of 10^−5^ M isoproterenol (ISO) or forskolin, a direct activator of adenylyl cyclase that bypasses the G-coupled protein receptors, in 1.0 mL of HBSS containing 1.0 mM IBMX (a cAMP phosphodiesterase inhibitor) at 37°C for 10 min. The reaction was then stopped by addition of 3 mL ice cold RPMI 1640 to the tubes. Samples then were centrifuged at 4°C for 8 min at 1,000 rpm, and the supernatants decanted. The cell pellets were resuspended in 0.5 mL 50 mM sodium acetate buffer. Splenocytes were lysed by two cycles of freezing (dry ice) and boiling (100°C water bath) for 5 min each. The samples were centrifuged for 10 min at 3,000 rpm to remove cellular debris, and the supernatants were collected and stored at −70°C until cAMP analysis by EIA according to the manufacturer's instructions.

### 2.7. Radioligand Binding Assays

Radioligand binding studies were performed on whole splenocytes from CFA- and saline-treated rats (*n* of 8 per group) on D21 after challenge. Binding studies were completed using the ligand (^125^I)cyanopindolol ((^125^I)CYP), a *β*-AR antagonist with equal affinity for the *β*_1_- and *β*_2_-AR subclasses. Spleen cells used for *β*-AR binding assays were resuspended to 5 × 10^6^ cells/mL RPMI 1640. (^125^I)CYP (2200 Ci/mMole) was diluted in 1% ethanol, 5 mM HCl, and 0.2% bovine serum albumin (BSA). Assays were performed in duplicate in 13 × 100 mm polypropylene tubes containing 1 × 10^6^ spleen cells with 8 concentrations of (^125^I)CYP ranging from 15.8 to 333 pM. Nonspecific binding was determined using parallel assays incubated in the presence of 10^−6^ M CGP-12177, a hydrophilic *β*-AR antagonist. Tubes were incubated at 37°C for 60 min in a shaking water bath (100 oscillation/min) to ensure that equilibrium was reached. The reaction was terminated with the addition of 3 mL of ice cold hypotonic buffer (3.8 mM KH_2_PO_4_, 16.2 mM K_2_HPO_4_, and 4 mM MnSO_4_) for 20 min to lyse the cells. The reaction mixture was filtered using a cell harvester (Brandel Corp., Gaithersburg, MD) and bound radioactivity collected on Whatman fiberglass filters (GF/B) (Brandel Corp., Gaithersburg, MD). Filters were washed with 16 mL (4 × 4 mL) of ice-cold Tris-EGTA buffer to remove the unbound radioligand. Filters were removed, placed in 12 × 75 mm tubes, and counted in a gamma counter (Whatman) at 82% efficiency.

Specific binding was defined as the difference between binding of the radioligand at each concentration in the absence or presence of 1 *μ*M propranolol. Nonspecific binding ranged from 8 to 10% of total binding. Receptor density (*B*_max⁡_) and affinity (*K*_*D*_) were determined using an iterative nonlinear regression curve fitting program, Prism, version 4, to a model of a single class of homogenous binding sites. Data were transformed into linear form by Scatchard analysis. Lines of best fit were generated using the *B*_max⁡_ and *K*_*D*_ to determine the *X* and *Y* axis intercepts. The maximal number of binding sites per cell was calculated based on simple stoichiometric assumptions (1 molecule of ligand binding to 1 receptor site) and expressed as sites/cell. 

### 2.8. p*β*_2_-AR and *β*_2_-AR Western Blots

Spleen and DLN cell pellets containing 5 × 10^6^ cells were lysed by addition of 1 mL of ice cold RIPA buffer (50 mM Tris 1% IGEPAL, 0.25% sodium deoxycholate, 150 mM sodium chloride, 1 mM EGTA, and 1 mM NaF; pH 7.4) containing a cocktail of protease inhibitors (Complete EDTA-free, Roche Diagnostics, Indianapolis, IN) and phosphatase inhibitor (Phosstop, Roche Diagnostics). Cell samples were placed on ice, vortexed every 10 min for 30 min, and then centrifuged at 14,000 rpm for 5 min. Supernatants were collected for western blot analysis and Bradford protein determination. Samples (20 *μ*g protein) were treated with reducing agent, denatured at 95°C for 5 min, and then cooled on ice. The samples were loaded onto a 4–12% acrylamide gel, ran at a constant 70 V through stacking, and then increased to 200 V for resolving of the protein bands. The proteins were transferred to polyvinylidene fluoride (PVDF) membranes at 400 mA for 1 h. The membranes were blocked for 1 h using Tris-buffered saline and Tween 20 (TBST, 10 mM Tris HCl, 100 mM NaCl, 0.1% Tween 20, pH 7.4)/5% milk and washed in TBST for 5 min (six times). The membranes were then placed in TBST/5% milk containing primary antibody (*β*_2_-AR_T_) (catalog number, SC569; 1 : 200), p*β*_2_-AR (Ser 345/346; catalog number, SC16718; 1 : 300), or p*β*_2_-AR (Ser 355/356; catalog number, SC16719R; 1 : 300) and then incubated overnight at 4°C. The blots were washed in TBST for 5 min (six times) at room temperature and then placed into TBST/5% milk containing biotinylated anti-rabbit (1 : 3,000) secondary antibody for 1 h. Next, the blots were washed in TBST for 5 min (six times) and then incubated with Supersignal Chemi Substrate (Pierce Chemical Company, Rockford, IL) for 5 min at room temperature then apposed to CL-Xposure Film (Pierce Chemical Company). Antibodies were stripped from the blots by applying Restore Stripping Buffer (Pierce Chemical Company) for 15 min at 37°C with gentle agitation. The blots were then washed twice in TBST for 10 min each and blocked for 1 h with TBST/5% milk. The blots were then reprobed for *β*-actin (1 : 5,000) as a loading control. Analysis of blots was completed using MCID 7.0 software (Image Research Inc., ON, Canada), and GraphPad PRISM software was used for data analyses. 

### 2.9. Cell Culture for Cytokine Production

Spleen and DLN cells suspended at a final concentration of 2 × 10^6^ cells/mL were plated into 24-well plates (Falcon, Oxnard, CA) and then placed in an incubator set at 7% CO_2_ and 37°C for 24 h. After 24 h, the supernatants were harvested and stored in the freezer at −80°C. ELISAs for IFN-*γ* were completed with standards run on each plate and according to the manufacturer's directions. Plates were read at 450 nm within 30 min with *λ* correction 570 nm using an ELISA plate reader (Ceres 900 HDI, Bio Tek Instruments Incorporated, Winooski, VT) and cytokine levels determined by comparing optical densities from a standard curve created using known concentrations of IFN-*γ* present on each plate. 

### 2.10. Statistical Analysis

#### 2.10.1. Disease Outcome Measures

 The average of the right and left foot pad measurements was obtained for each animal, and then these individual means were averaged within each group, expressed as a mean ± standard error of the mean (SEM), and analyzed using a two-way analysis of variance (ANOVA) with repeated measures. Significant ANOVA (*P* < 0.05) values were subjected to Bonferroni posthoc testing. The sum of the radiographic scores for the hind limbs was averaged within the treatment groups and expressed as a group mean ± SEM and subjected to Kruskal-Wallis statistical analysis (nonparametric statistic equivalent to an ANOVA; *P* < 0.05) followed by Dunn posthoc testing. 

#### 2.10.2. cAMP Production, *β*_2_-AR Binding Assays, Cytokine Production, and *β*_2_-AR Protein Levels

 Replicated individual optical densities (OD) from *β*_2_-AR western blots normalized to *β*-actin, *β*_2_-AR *K*_*D*_ and *B*_max⁡_, or fmol cAMP/2 × 10^6^ cells were averaged for each animal and then for each treatment group. Data were expressed as a mean ± SEM and analyzed using one-way ANOVA with Bonferroni posthoc testing. OD obtained for p*β*_2_-AR_PKA_ and p*β*_2_-AR_GRK_ were also expressed as a ratio of the *β*_2_-AR_T_ OD. IFN-*γ* concentrations from duplicate wells were averaged, group means were calculated, and the data are expressed as a mean in pg/mL. Significant differences between treatment groups for mean *β*_2_-AR, p*β*_2_-AR_PKA_ and p*β*_2_-AR_GRK_ OD and the ratios for p*β*_2_-AR_PKA_ and p*β*_2_-AR_GRK_ to *β*_2_-AR_T_ were determined using one-way ANOVA (*P* < 0.05) followed by Bonferroni post hoc analysis. Differences between treatment groups for production of IFN-*γ* were determined using a Student's *t*-test (*P* < 0.05). Only data where significant differences (*P* < 0.1) between treatment groups were observed will be described.

## 3. Results

### 3.1. Disease Severity

**Figure 1:**
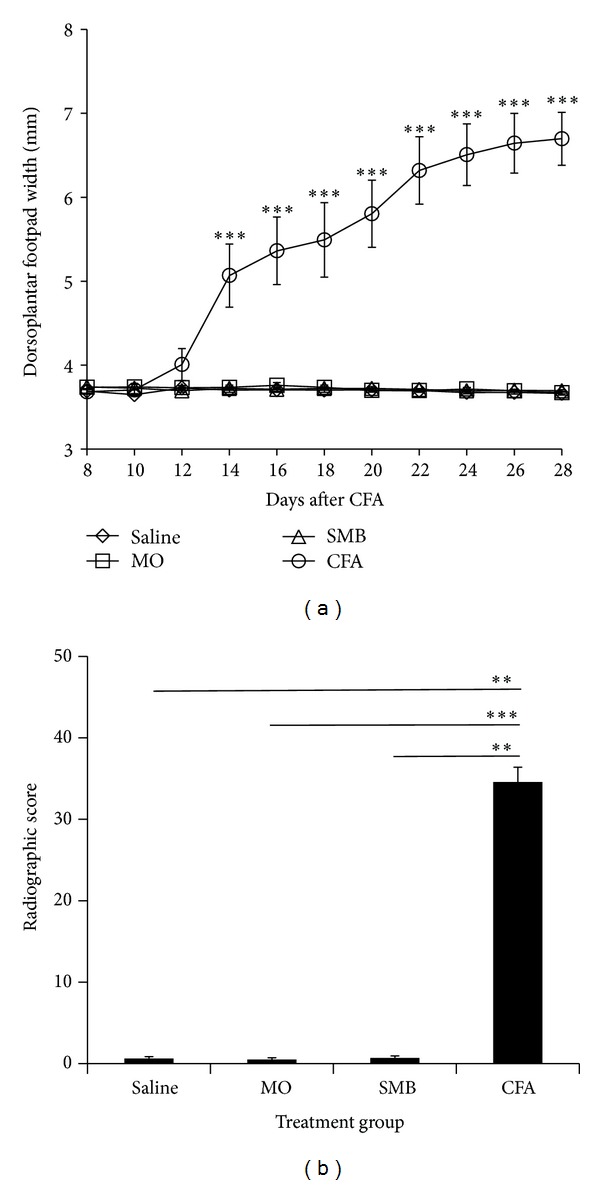
Mean hind limb dorsoplantar widths (a) and radiographic scores (b) for rats treated with CFA, SMB, MO, or Saline. (a) A significant increase in mean dorsoplantar widths, indicative of soft tissue swelling, was observed in CFA (O)-challenged rats compared with groups treated with SMB (Δ), MO (□) or Saline (*◊*) between D12–28 after CFA immunization. No differences were observed in dorsoplantar widths between SMB-, MO- or Saline-treated rats. Values represent the mean dorsoplantar width in millimeters (mm) ± SEM with an *n* of 8 rats per treatment group. Statistics: repeated measures two-way ANOVA with Bonferroni multiple comparison tests (***P < 0.001). (b) Rats treated with CFA had significantly higher radiographic scores compared with rats treated with SMB, MO, or Saline on D28 post-immunization. No differences were observed in radiographic scores between SMB-, MO- or Saline-treated rats. Data are expressed as the mean radiographic score ± SEM with an *n* of 8 rats per treatment group. Statistics: Kruskal-Wallis analysis followed by Dunn post hoc testing (**P < 0.01; ***P < 0.001).

Animals challenged with saline, SMB, or MO developed no clinical signs of arthritis throughout the duration of the experiment (Figure 1(a)). Erythema was apparent in CFA-challenged rats by D8 after CFA challenge. Dorsoplantar foot pad width increased in the CFA-challenged animals, reaching significance (*P* < 0.0001) between D12–14 compared with Saline-, SMB-, or MO-treated rats (Figure 1(a)) (CFA versus Saline, CFA versus SMB, and CFA versus MO: *P* < 0.0001 at each time point). Dorsoplantar foot pad widths of CFA-challenged rats continued to increase through D21–24 (severe disease) and then stabilized through D28 (chronic disease). Radiographic analysis of the ankle joints (Figure 1(b)) revealed significant differences between treatment groups (*P* < 0.0004). CFA-challenged rats had increased soft tissue swelling, bone loss, periosteal bone formation, narrowing of their joint spaces, and reduced bone density by D28 after CFA challenge compared with the Saline- (*P* < 0.01), MO- (*P* < 0.001), or SMB- (*P* < 0.01) challenged rats. 

### 3.2. *β*-AR-Stimulated cAMP Production in Spleen Cells

**Figure 2:**
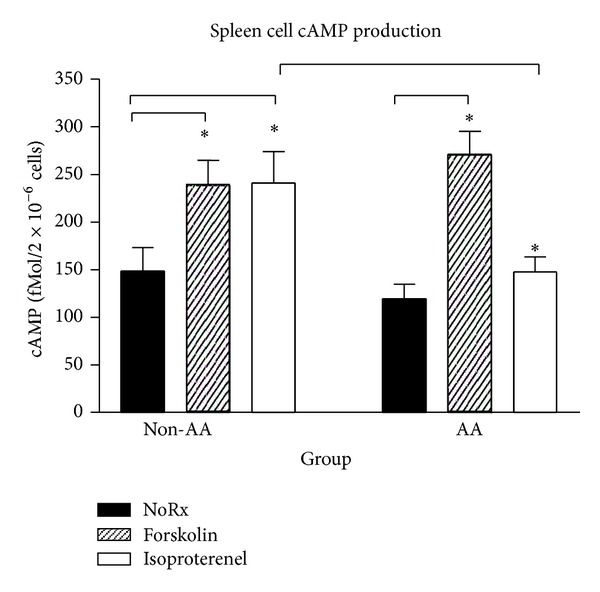
Forskolin and isoproterenol elevated cAMP production compared with untreated spleen cells *in vitro* in saline-treated non-AA rats. In contrast, forskolin, but not isoproterenol treatment, increased cAMP production compared with untreated splenocytes in AA rats. The lower cAMP production observed in splenocytes treated with isoproterenol in AA rats was also significantly different compared with non-AA rats. Data are expressed as a mean ± SEM with an *n* of 8 rats per treatment group. Statistics: one-way ANOVA with Bonferroni multiple comparison tests (*P < 0.05).

On D21, unstimulated splenocytes from Saline- and CFA-challenged rats contained similar baseline levels of cAMP. Treatment with forskolin (Figure 2) significantly increased splenocyte cAMP production in nonarthritic and CFA-treated rats from basal levels (40 and 54%, resp., *P* < 0.05). Similarly, isoproterenol-stimulated cAMP production rose 40% (*P* < 0.05) higher than in unstimulated spleen cells from nonarthritic rats. In contrast, adding isoproterenol to the spleen cell suspension from arthritic rats had no effect on cAMP production. 

### 3.3. Receptor Binding Assays

**Figure 3:**
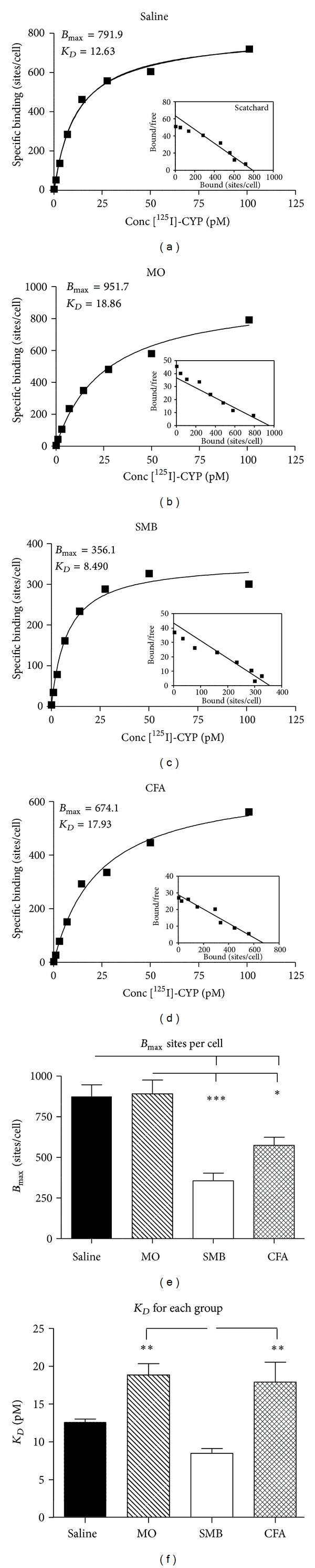
Specific binding and Scatchard plots of (−)-(^125^I)cyanopindolol (^125^ICYP) in whole spleen cells from rats treated with (a) Saline, (b) MO, (c) SMB, and (d) CFA. Spleen cells were incubated under equilibrium binding conditions at 37°C with ^125^ICYP (15.8–333 pM) for 60 min, then the reaction was stopped, and the radioactivity was quantified by gamma scintillation counting. Binding assays were run in duplicate. Specific binding (sites/cell) and inset Scatchard plots (bound/free) represent mean values obtained from 8 rats in each treatment group. (e)-(f). The mean density of *β*-AR (*B*_max⁡_) expressed as sites/cell (e) and *K*_*D*_ (f) on spleen cells from Saline-, MO-, SMB-, and CFA-treated rats. (e) The number of *β*_2_-AR sites per splenocyte is significantly decreased in SMB- and CFA-treated rats compared with both Saline- and MO-treated rats. (f) The mean *K*_*D*_ was increased in MO- and CFA-treated rats compared with rats treated with SMB. Mean values were calculated for the *B*_max⁡_ and *K*_*D*_ determined from specific binding curves generated for each rat from each treatment group. Data are expressed as a mean *B*_max⁡_ or *K*_*D*_  ± SEM with an *n* of 8 rats per treatment group. Statistics: one-way ANOVA with Bonferroni multiple comparison tests (*P < 0.05; **P < 0.01; ***P < 0.001).

On D21 after treatment with Saline, MO, SMB, or CFA, saturation isotherms obtained from binding experiments with splenocytes are shown in Figures 3(a)–3(d). Binding of the radioligand was rapid, saturable, and of high affinity in all treatment groups and at each time point. Specific binding was greater than 90% of the total binding at near saturating radioligand concentrations. Scatchard plots are shown in the insets of Figures 3(a)–3(d). ANOVA indicated significant differences in the mean *B*_max⁡_ between treatment groups (*P* < 0.0002). Mean *B*_max⁡_ (Figure 3(a)) was significantly reduced in both SMB- and CFA-challenged rats compared with the Saline and MO treatment groups (SMB versus Saline or MO, *P* < 0.001; CFA versus Saline or MO, *P* < 0.05). The mean *K*_*D*_ differed significantly between treatment groups (*P* < 0.0009). *K*_*D*_ was significantly higher (*P* < 0.01) for MO- or CFA-treated compared with SMB-treated rats. There was a trend (*P* < 0.1) for an increased mean *K*_*D*_ in splenocytes from MO- and CFA- compared with Saline-treated rats. 

### 3.4. *β*_2_-AR Expression Is Reduced in Splenocytes, but Not in DLN Cells in Established AA

**Figure 4:**
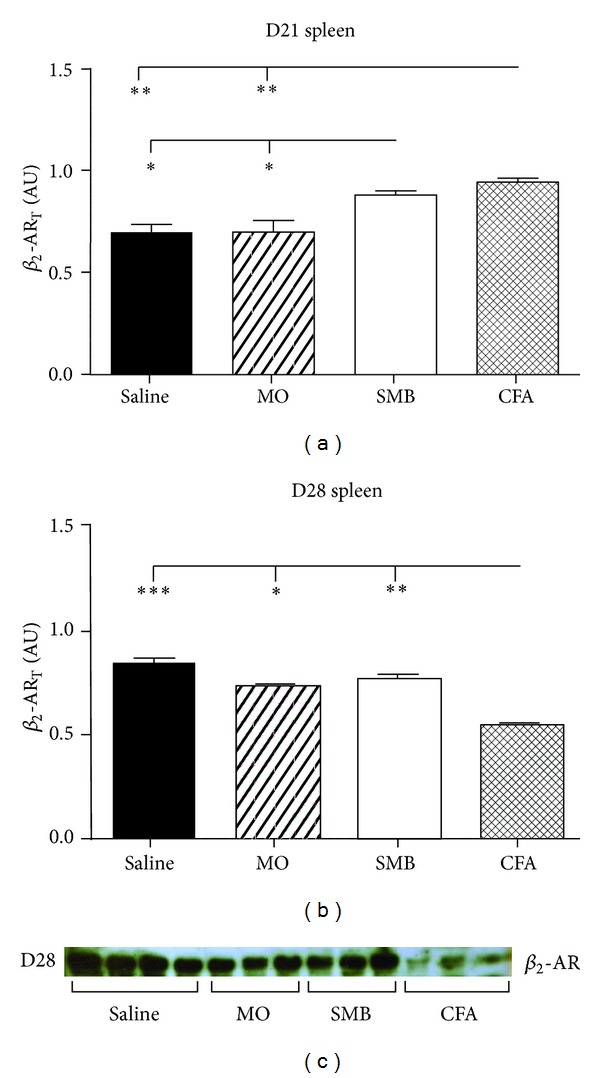
Total *β*_2_-AR expression in splenocytes from AA and non-AA rats at (a) D21 and (b) D28 after immune challenge. (a) Expression of *β*_2_-AR was increased on D21 in splenocytes from SMB- and CFA-treated rats compared with Saline- and MO-treated rats. (b) In contrast, on D28, *β*_2_-AR expression was significantly decreased in CFA-treated compared with all other treatment groups. (c) Representative immunoblot depicting *β*_2_-AR expression in splenocytes from each treatment group on D28 after CFA challenge. Spleen cells were harvested, lysed, and proteins resolved by SDS-PAGE. The data were normalized to *β*-actin. Cellular extracts were probed with a *β*_2_-AR receptor antibody to determine *β*_2_-AR expression and quantified by densitometry. Each bar represents the mean optical density ± SEM with an *n* of 8 rats per treatment group. Statistics: one-way ANOVA with Bonferroni multiple comparison tests. (*P < 0.05; **P < 0.01; ***P < 0.001).

**Figure 5:**
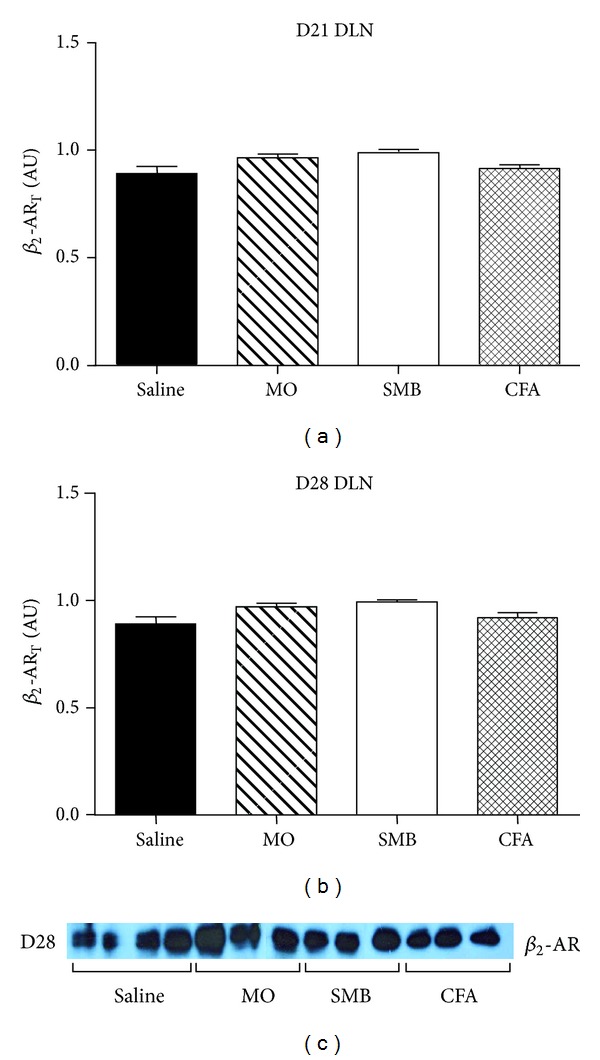
Total *β*_2_-AR expression in DLN cells from AA and non-AA rats at (a) D21 and (b) D28 after immune challenge ((a)–(c)). In contrast to splenocytes, expression of *β*_2_-AR was unchanged in DLN cells from CFA-, SMB-, and MO-challenged rats on D21 or D28 compared with Saline-treated controls. DLN cells were harvested, lysed, and proteins resolved by SDS-PAGE. The data were normalized to *β*-actin. Cellular extracts were probed with a *β*_2_-AR receptor antibody, and *β*_2_-AR expression was quantified by densitometry. An example of the western blots at D28 is shown in (c). Each bar represents the mean optical density ± SEM with an *n* of 8 rats per treatment group. A one-way ANOVA with Bonferroni multiple comparison tests was used to determine statistically significant differences between treatment groups.

To determine if *β*_2_-AR expression is altered in animals with established AA, splenocyte *β*_2_-AR expression was assessed by western blot (Figures 4(a)–4(c)). On D21, ANOVA revealed significant differences (*P* < 0.0005) in splenocyte *β*_2_-AR density between treatment groups (Figure 4(a)). Immune challenge with CFA (*P* < 0.01) or SMB (*P* < 0.05) significantly elevated the splenocyte *β*_2_-AR density compared with Saline or MO treatment. There also was a significant difference (*P* < 0.005) in splenocyte *β*_2_-AR density between treatment groups on D28 (Figure 4(b)). Spleen cells from CFA-challenged rats had significantly decreased *β*_2_-AR density compared with Saline (35%, *P* < 0.001), MO (26%, *P* < 0.05), or SMB (29%, *P* < 0.01) treatment groups. A representative immunoblot depicting *β*_2_-AR expression in splenocytes from each treatment group on D28 after CFA challenge is shown in Figure 4(c). No difference in *β*_2_-AR expression in DLN cells was observed between any of the treatment groups at either time point after CFA challenge (Figures 5(a)–5(c)). 

### 3.5. AA Reduces Phosphorylation of Splenocyte *β*_2_-AR at Ser 345-346 (PKA Site)

**Figure 6:**
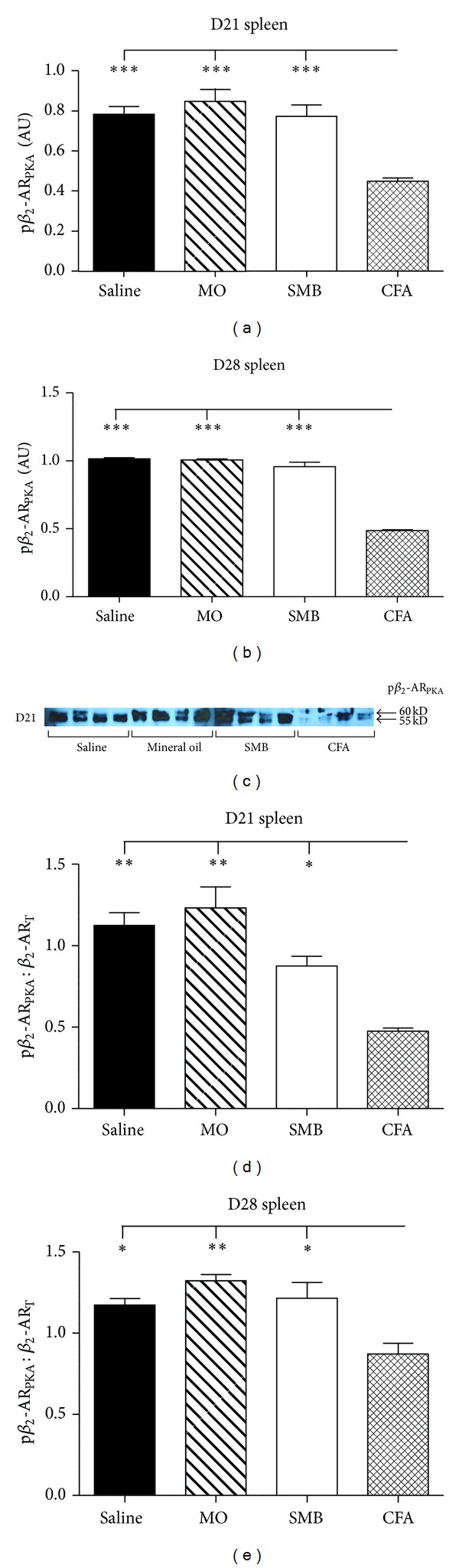
Expression of PKA-phosphorylated-*β*_2_-AR (p*β*_2_-AR_PKA_) in splenocytes from arthritic and nonarthritic control rats. ((a)–(e)). Expression of p*β*_2_-AR_PKA_ in splenocytes was reduced in CFA- compared with SMB-, Saline-, and MO-challenged rats D21 (a) and D28 (b) after immune challenge. Expression of p*β*_2_-AR_PKA_ in splenocytes from AA and non-AA rats at D21 (d) and D28 (e) after immune challenge was normalized to total receptor (*β*_2_-AR_T_) levels. *β*_2_-AR_PKA_/*β*_2_-AR_T_ was reduced in CFA-challenged rats compared with SMB-, MO-, and Saline-treated rats at both time points examined, indicating a disease-specific effect. Spleen cells were harvested, lysed, and total protein collected, then resolved by SDS-PAGE. The data were normalized to *β*-actin. Cellular extracts were probed with an antibody against phosphorylated Ser345, Ser346 of the *β*_2_-AR and quantified by densitometry. An example of the western blots at D21 is shown in (c). Each bar represents the mean optical density ± SEM (a)-(b) or mean p*β*_2_-AR_PKA_ normalized to *β*_2_-AR_T_  ± SEM (d)-(e) with an *n* of 8 rats per treatment group. Data were analyzed using one-way ANOVA with Bonferroni posthoc testing (*P < 0.05; **P < 0.01; ***P < 0.001).

To determine if the lack of isoproterenol-induced cAMP response observed in splenocytes from rats with established arthritis could be due to p*β*_2_-AR_PKA_ with subsequent uncoupling of the receptor to Gs proteins, splenocyte p*β*_2_-AR_PKA_ expression was assessed using western blots. On D21 and D28 after immunization, significant main effects of treatment (*P* < 0.0001 at both time points) were observed (Figures 6(a)–6(c)). On D21, expression of p*β*_2_-AR_PKA_ in splenocytes from CFA-challenged rats was significantly reduced (*P* < 0.001) by 43, 47, and 42% compared with Saline-, MO-, and SMB-challenged rats, respectively (Figure 6(a)). Similarly, on D28, p*β*_2_-AR_PKA_ in splenocytes from CFA-challenged rats were significantly reduced (*P* < 0.001) by 52, 52, and 49% compared with Saline-, MO-, and SMB-challenged rats, respectively (Figure 6(b)). A representative immunoblot depicting p*β*_2_-AR_PKA_ expression in splenocytes from each treatment group on D28 after CFA challenge is shown in Figure 6(c).

ANOVA revealed significant differences in the p*β*_2_-AR_PKA_ on D21 (*P* < 0.0001) and D28 (*P* < 0.0042), when expressed as a p*β*_2_-AR_PKA_/*β*_2_-AR_T_ (Figures 6(d) and 6(e)). There was a trend (*P* < 0.1) towards lower p*β*_2_-AR_PKA_/*β*_2_-AR_T_ expression for SMB- compared with Saline- or MO-treated on D21 (Figure 6(d)). p*β*_2_-AR_PKA_/*β*_2_-AR_T_ were significantly reduced by 58 (*P* < 0.01), 61 (*P* < 0.01), and 48% (*P* < 0.05) in arthritic CFA- compared with Saline-, MO-, and SMB-treated rats on D21, respectively. Similarly, p*β*_2_-AR_PKA_/*β*_2_-AR_T_ were significantly decreased on D28 by 26 (*P* < 0.05), 34 (*P* < 0.01), and 28% (*P* < 0.05) in arthritic rats compared with the Saline, MO, and SMB treatment groups, respectively (Figure 6(e)).

### 3.6. Spleen Cell p*β*_2_-AR at Ser 355-356 (GRK Site) Is Increased in AA Rats

**Figure 7:**
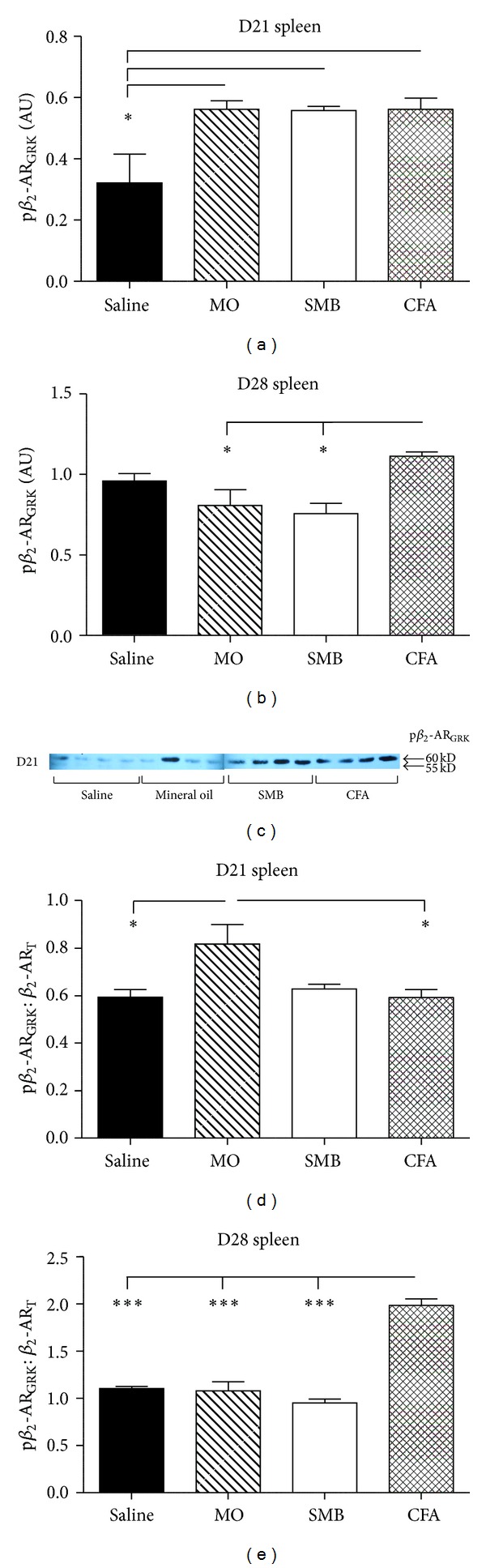
Expression of GRK phosphorylated-*β*_2_-AR (p*β*_2_-AR_GRK_) in splenocytes from arthritic and nonarthritic control rats. (a) Expression of p*β*_2_-AR_GRK_ in splenocytes was increased in CFA-, SMB-, and MO-challenged rats compared with Saline-treated rats on D21. (b) By D28, p*β*_2_-AR GRK expression in spleen cells from SMB- and MO-challenged rats was reduced compared with CFA-challenged rats. No difference in p*β*_2_-AR_GRK_ expression between CFA- and Saline-treated rats was observed. (d) Expression of p*β*_2_-AR_GRK_ in splenocytes from AA and non-AA rats normalized to *β*_2_-AR_T_ levels. MO-treated rats expressed greater p*β*_2_-AR_GRK_/*β*_2_-AR_T_ levels compared with Saline- and CFA-treated rats on D21 after immune challenge. (e) On D28 after challenge, p*β*_2_-AR_GRK_/*β*_2_-AR_T_ was significantly increased in CFA-challenged rats compared with all other treatment groups. Splenocytes were harvested, lysed, and proteins resolved by SDS-PAGE. Cellular extracts were probed with an antibody against phosphorylated Ser355/Ser356 of the *β*_2_-AR. (c) Western blot shown is representative of the blots within each treatment group. The data were normalized to *β*-actin. Data are expressed as a mean p*β*_2_-AR_GRK_  ± SEM (a)-(b) or mean p*β*_2_-AR_GRK_ normalized to *β*_2_-AR_T_  ± SEM (d)-(e) with an *n* of 8 rats per treatment group. Data were analyzed using one-way ANOVA with Bonferroni posthoc testing (*P < 0.05; **P < 0.01; ***P < 0.001).

Analysis of western blots revealed significant treatment effects on expression of p*β*_2_-AR_GRK_ for both D21 and D28 after immune challenge (*P* < 0.0154 and *P* < 0.0134, resp.; Figures 7(a)–7(c)). At D21, p*β*_2_-AR_GRK_ in splenocytes from rats challenged with MO, SMB, or CFA were significantly increased (43%; *P* < 0.05) compared with Saline-treated rats (Figure  7(a)). On day 28, p*β*_2_-AR_GRK_ trended towards being reduced (*P* < 0.1) in SMB- compared with Saline-treated rats (Figure 7). Only p*β*_2_-AR_GRK_ in splenocytes from arthritic rats were significantly increased compared with MO (28%) and SMB (32%; *P* < 0.05). A representative immunoblot depicting p*β*_2_-AR_GRK_ expression in splenocytes from each treatment group on D28 after CFA challenge is shown in Figure 7(c).

Western blot data for p*β*_2_-AR_GRK_ were also expressed as p*β*_2_-AR_GRK_/*β*_2_-AR_T_ (Figures 7(d) and 7(e)). ANOVA revealed significant differences in the p*β*_2_-AR_GRK_/*β*_2_-AR_T_ on D21 (*P* < 0.0181) and D28 (*P* < 0.0001) (Figure 7(d)). On D21, p*β*_2_-AR_GRK_/*β*_2_-AR_T_ were elevated 27% in MO- compared with Saline- or CFA-treated rats (*P* < 0.05) (Figure 7(d)). In MO-treated rats, this ratio trended toward an increase compared with SMB-treated rats (23%, *P* < 0.1). p*β*_2_-AR_GRK_/*β*_2_-AR_T_ in CFA-treated rats significantly increased (*P* < 0.001) compared with Saline- (44%), MO- (46%) or SMB- (52%) treated rats on D28 after immune challenge (Figure 7(e)).

### 3.7. Phosphorylation of DLN Cell *β*_2_-AR at Ser 345-346 (PKA Site) Increased at Peak Disease

**Figure 8:**
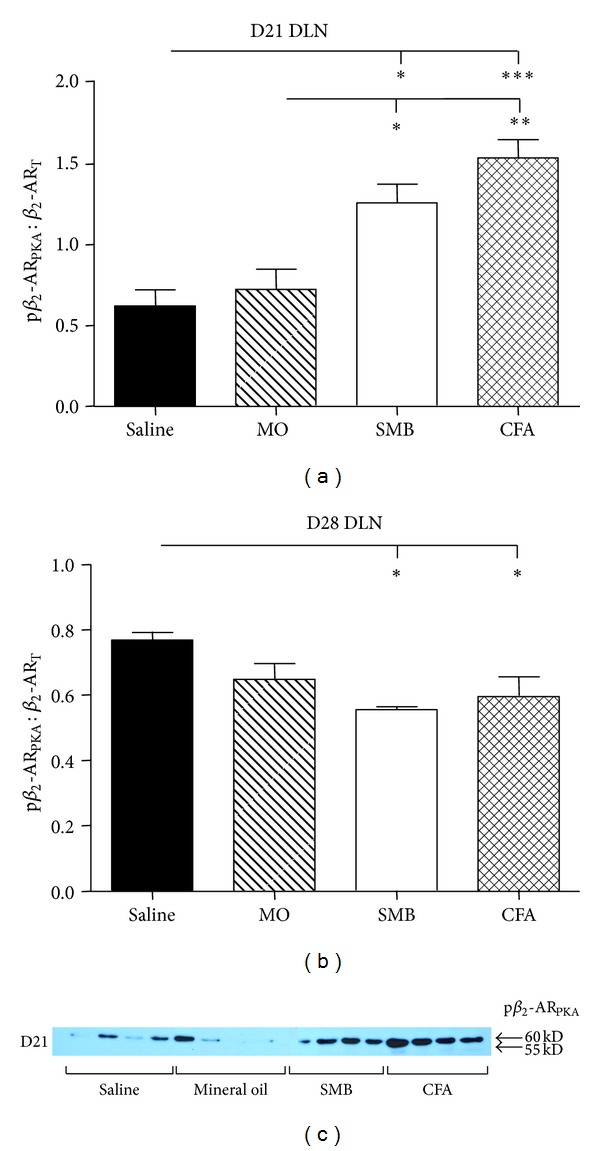
Expression of PKA-phosphorylated-*β*_2_-AR (p*β*_2_-AR_PKA_)/*β*_2_-AR_T_ in DLN cells from rats challenged with CFA, SMB, or MO compared with Saline-treated controls. Levels of p*β*_2_-AR_PKA_/*β*_2_-AR_T_ were not significantly different in Saline- and MO-treated rats on D21 (a) or D28 (b). In contrast, p*β*_2_-AR_PKA_/*β*_2_-AR_T_ in DLN cells from CFA- and SMB-challenged rats were significantly increased on D21, but significantly decreased on D28 compared with Saline- or MO-treated rats. DLN cells were harvested, lysed, and total protein collected and resolved by SDS-PAGE. Cellular extracts were probed with an antibody against the phosphorylated Ser345, Ser346 of the *β*_2_-AR to determine p*β*_2_-AR_PKA_ expression and quantified by densitometry. A western blot is shown (c) that is representative of the blots seen within each treatment. The data were normalized to *β*-actin, then p*β*_2_-AR_PKA_ was normalized to *β*_2_-AR_T_ levels. Each bar represents the mean p*β*_2_-AR_PKA_/*β*_2_-AR_T_  ± SEM with an *n* of 8 rats per treatment group. Data were analyzed using one-way ANOVA with Bonferroni posthoc testing (*P < 0.05; **P < 0.01; ***P < 0.001).

Expression of p*β*_2_-AR_PKA_ was also assessed in DLN cells from arthritic and nonarthritic treatment groups (Figures 8(a) and 8(b)). ANOVA revealed significant differences in the p*β*_2_-AR_PKA_/*β*_2_-AR_T_ on D21 (*P* < 0.0002) and D28 (*P* < 0.0136). On day 21, p*β*_2_-AR_PKA_/*β*_2_-AR_T_ in DLN cells increased in SMB-treated rats compared with Saline or MO treatment (*P* < 0.05) and in CFA-challenged rats compared with Saline- or MO-treated rats (*P* < 0.001 or *P* < 0.01, resp.) (Figure 8(a)). In contrast, p*β*_2_-AR_PKA_/*β*_2_-AR_T_ in DLN cells from CFA- and SMB-challenged rats were decreased (*P* < 0.05) on D28 compared with Saline-treated rats (Figure 8(b)). 

### 3.8. Phosphorylation of *β*_2_-AR at Ser 355-356 (GRK Site) in DLN Cells Is Increased in AA Rats

**Figure 9:**
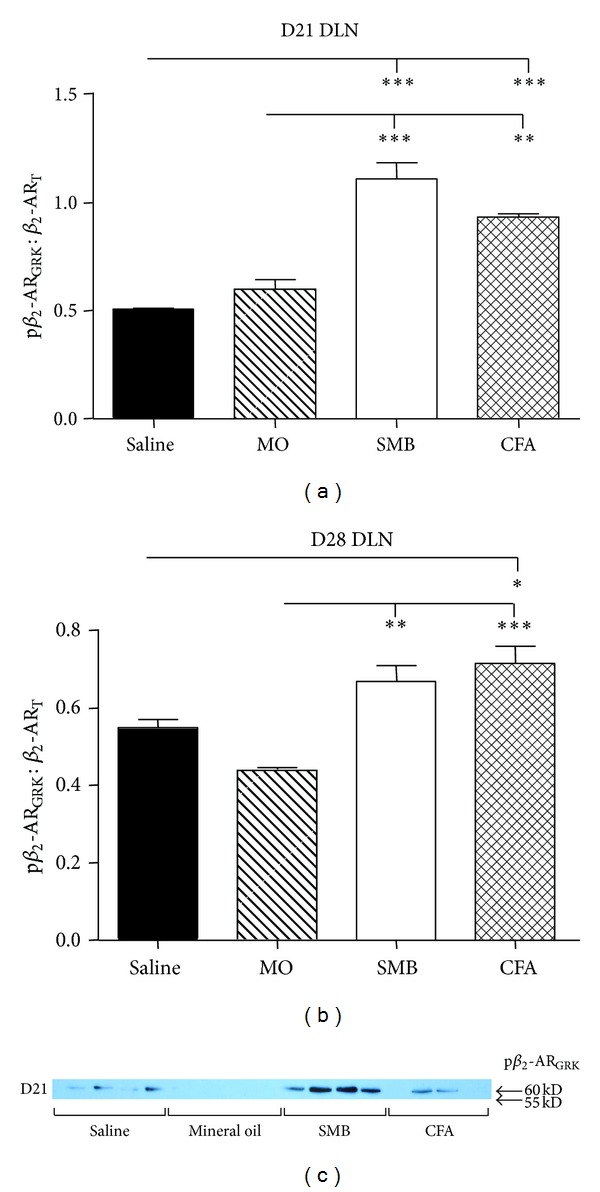
Expression of the p*β*_2_-AR_GRK_/*β*_2_-AR_T_ in DLN cells from rats challenged with CFA, SMB, or MO compared with Saline-treated controls on D21 (a) and D28 (b). No difference in p*β*_2_-AR_GRK_/*β*_2_-AR_T_ was observed between the MO- and Saline-treatment groups at either time point. Expression of p*β*_2_-AR_GRK_/*β*_2_-AR_T_ was increased in CFA-, SMB- compared with MO-challenged and Saline-treated rats on D21 and D28. DLN cells were harvested, lysed, and proteins resolved by SDS-PAGE. Cellular extracts were probed with an antibody against GRK phosphorylated Ser355/Ser356 of the *β*_2_-AR. A western blot is shown (c) that is representative of the blots seen within each treatment. The data were normalized to *β*-actin, and p*β*_2_-AR_GRK_ expression was normalized to *β*_2_-AR_T_. Data are expressed as a mean p*β*_2_-AR_GRK_/*β*_2_-AR_T_  ± SEM with an *n* of 8 rats per treatment group. Data were analyzed using one-way ANOVA with Bonferroni posthoc testing (**P < 0.01; ***P < 0.001).

Analysis of western blots revealed significant treatment effects on expression of p*β*_2_-AR_GRK_/*β*_2_-AR_T_ for both D21 and D28 after immune challenge (*P* < 0.0001 and *P* < 0.0004, resp.; Figures 9(a)–9(c)). p*β*_2_-AR_GRK_/*β*_2_-AR_T_ was significantly increased in SMB- and CFA-challenged rats compared with either the Saline- or MO-treated rats on D21 (SMB versus Saline or MO, *P* < 0.001; CFA versus Saline, *P* < 0.001 or MO, *P* < 0.01; Figure 9(a)). On D28, p*β*_2_-AR_GRK_/*β*_2_-AR_T_ significantly increased (*P* < 0.05 or *P* < 0.001, resp.) in CFA-challenged rats compared with either the Saline- or MO-treated rats (Figure 9(b)). p*β*_2_-AR_GRK_/*β*_2_-AR_T_ also significantly (*P* < 0.01) increased in SMB- compared with MO-treated rats. There was a trend (*P* < 0.1) for a higher ratio in SMB- compared with Saline-treated rats. 

### 3.9. Terbutaline Treatment Differentially Affected IFN-*γ* Production in Spleen and DLN Cells

**Figure 10:**
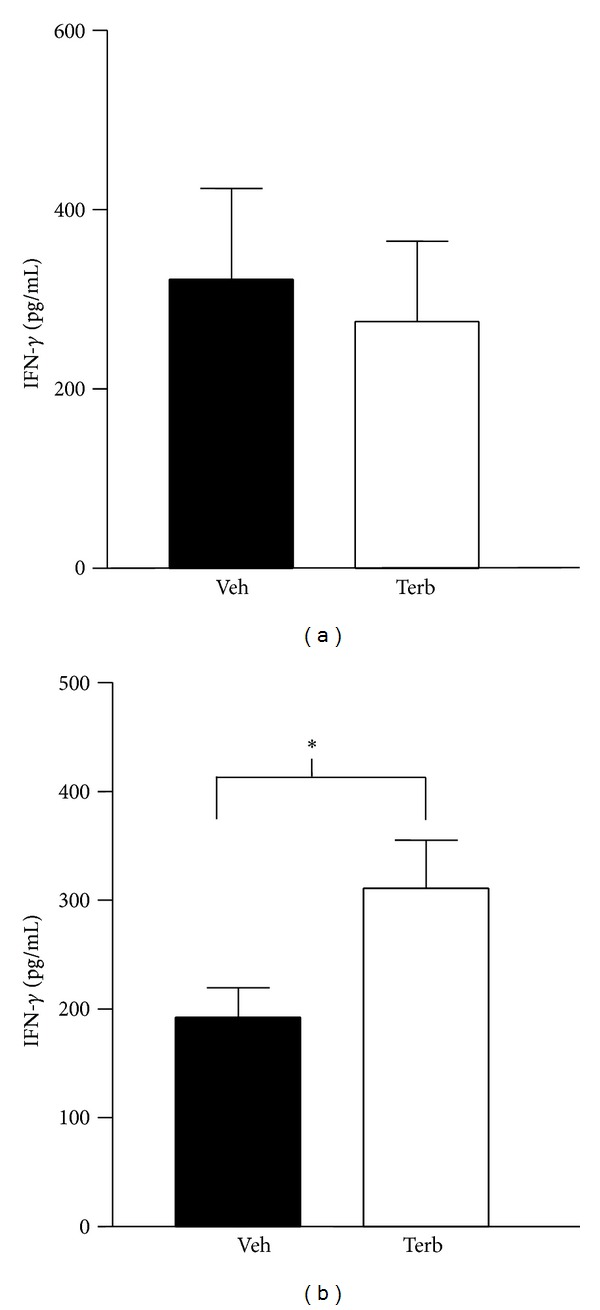
The effects of twice-daily treatment with terbutaline (Terb) (total of 1200 *μ*g/day i.p.) or vehicle (Veh) in arthritic rats from D12–D28 post-CFA challenge on *ex vivo* IFN-*γ* secretion by splenocytes (a), and draining lymph node (DLN) cells (b). Terb had no effect on IFN-*γ* secretion by cultured spleen cells (a), but significantly increased (*P < 0.05) IFN-*γ* production by DLN cells. Data are expressed as means ± SEM (*n* = 8/group) and analyzed using a Student's *t*-test.

Figure 10 shows the *ex vivo* secretion of IFN-*γ* in spleen (a) and DLN (b). IFN-*γ* production did not differ significantly between spleen cells obtained from arthritic rats treated with terbutaline compared with arthritic rats treated with the vehicle (Figure 10(a)). In contrast, terbutaline treatment in arthritic rats significantly increased DLN cell IFN-*γ* production compared with vehicle treatment (Figure 10(b); *P* < 0.05).

## 4. Discussion

In the present study, we have determined the effects of CFA, and its components, on *β*_2_-AR expression and signaling in immunocytes from lymphoid organs that mediate disease processes for RA. We report that in AA, during severe disease, *β*-AR agonists fail to induce cAMP production in splenocytes. Loss of *β*-AR-induced cAMP is accompanied by reduced affinity and expression of *β*-AR, indicative of receptor desensitization and downregulation. p*β*_2_-AR_PKA_ and p*β*_2_-AR_GRK_ are strikingly different in splenocytes and DLN cells from AA rats. In splenocytes, p*β*_2_-AR_PKA_ is reduced regardless of the time point examined, while in DLN cells, p*β*_2_-AR_PKA_ is increased during severe AA and reduced during chronic disease. Similarly, in splenocytes, p*β*_2_-AR_GRK_ is unchanged and increased with severe and chronic disease stages, respectively, but increased in DLN cells during both disease stages. Differential p*β*_2_-AR support disparate regulation of spleen and DLN cell functions by the SNS in AA rats. These findings are consistent with previous findings from our lab [41] and in the present study, with the findings from *in vivo* terbutaline treatment showing increased IFN-*γ* production by DLN cells, but no change in splenocyte IFN-*γ* production *ex vivo*.

In the present study, basal levels of cAMP were similar in AA and nonarthritic rats, but *β*-AR stimulation failed to alter cAMP levels in splenocytes from AA rats during peak disease severity (D21) compared with controls. Synthesis of cAMP was not impaired, because forskolin induced intracellular cAMP production in AA rats. Since *β*_2_-AR-Gs protein coupling is required for adenylyl cyclase-induced cAMP production, these findings support an uncoupling of *β*-ARs to Gs proteins in splenocytes from AA rats. Loss of catecholamine-induced cAMP production indicates altered receptor function and is supported by findings from the receptor binding studies. Receptor affinity (1/*K*_*D*_) was reduced in splenocytes from AA rats by D21 after CFA challenge compared with controls (Saline and SMB). Receptor affinity was also decreased in MO-treated controls, indicating that the MO component of CFA induced this effect. *B*_max⁡_ values were reduced in splenocytes from SMB and AA compared with Saline- and MO-treated rats. The effects of SMB treatment on *β*-AR density support a role for the bacterial cell wall component of CFA in this response. Collectively, our data indicate that splenocyte *β*_2_-ARs are desensitized and downregulated in severe AA, effects that are mediated via MO and SMB, respectively. 

Western blots revealed an increase in *β*_2_-AR_T_ expression (membrane and intracellular) in splenocytes obtained from AA rats on D21, an effect also observed in SMB-treated controls. In contrast, our binding data show reduced cell surface expression of *β*_2_-ARs, suggesting increased internalization of *β*_2_-ARs. By D28, *β*_2_-ARs were decreased in AA rats compared with all controls, indicating a disease-specific internalization and degradation of receptors between D21 and D28. In contrast, *β*_2_-ARs levels returned to baseline in SMB-treated rats at D28, supporting recycling of *β*_2_-AR to the membrane or *β*_2_-AR synthesis keeps pace with degradation.

Others have reported reduced *β*-AR agonist-induced cAMP and *β*-AR densities in PBMC and B cells in RA patients that negatively correlate with systemic disease activity [9, 43]. In contrast, *β*-AR agonist-induced cAMP do not differ in CD4+ and CD8+ T cells and are increased in PBMCs in RA patients compared with healthy controls in other studies [13, 43]. These seemingly inconsistent findings may be explained, in part by, the duration of *β*_2_-AR stimulation, use of membranes versus whole cells, and/or reported increase in the incidence of polymorphisms in the *β*_2_-AR in RA patients. RA patients have a greater incidence of the Arg^16^Gly polymorphism in the *β*_2_-AR compared with healthy controls, which in association with HLA-DR alleles imparts a greater risk for developing RA [44]. Further, these polymorphisms modulate the age of disease onset, with additional polymorphisms at Gln^27^Glu and Thr^164^Ile imparting an increased risk for developing arterial hypertension in RA patients [44]. Ahles et al. [45] have demonstrated that the Arg^16^Gly polymorphism imparts differential signaling of *β*_2_-ARs. Similar to Arg^16^ encoded receptors, the polymorphisms Arg^16^→Gly or Gln^27^→Glu is recruited to the cell membrane to a similar extent with similar ligand binding affinities. They also have similar activation and deactivation kinetics after a single stimulation with an *β*-AR agonist. However, receptors encoded by Arg^16^, Arg^16^Gly, and Gln^27^Glu *β*_2_-AR genes display different activation kinetics after repeat activation that result in differential efficacies to generate cAMP [45] reviewed in [46]. The Arg^16^Gly polymorphism result in faster, more persistent, and more effective downstream signaling via cAMP regardless of its association with Gln^27^ or Glu^27^. In contrast, the Arg^16^Gln^27^ variant demonstrates slower activation kinetics and reduced cAMP production, an effect independent of receptor internalization, as this did not differ among the three *β*_2_-AR variants. Enhanced activation of the Arg^16^GLy is coupled with greater phosphorylation of the receptor by GRK-2 and a more rapid recruitment of *β*_2_-arrestin to *β*_2_-AR, which is required for enhanced activation kinetics. Once *β*_2_-AR is stimulated the altered activation kinetics is independent of activation by G proteins. Thus, reported inconsistencies in lymphocyte *β*_2_-AR-induced cAMP between RA and healthy controls may result from differences in duration and repetitiveness of receptor stimulation.

It is not known whether different rodent strains also have variants in genes encoding *β*_2_-AR. if they do, they could explain differences in strain susceptibility to induction of inflammatory arthritis. If so, these strains could provide models for understanding the contribution of *β*_2_-AR polymorphisms to disease onset and severity in RA patients. An understanding of the physiological and clinical relevance of *β*_2_-AR variants could substantially enhance our understanding of mechanisms for differences among experimental subjects and patients in their response to *β*_2_-AR agonists reviewed in [47]. Collectively, our findings in AA and reported findings in RA suggest that complex site-and cell-specific changes in signal transduction and *β*_2_-AR polymorphisms impart different catecholamine-induced effects on lymphocyte functions in RA.

Since the SNS dampens inflammation and cell-mediated responses, impaired *β*-AR signaling in splenocytes of AA rats is expected to promote disease processes. However, previous studies from our group [5] and others [6] have shown that *β*-AR agonists reduce disease severity if administered from disease onset through severe disease. Disruption of *β*-AR signaling in splenocytes after disease onset suggests that the attenuating effects of *β*-agonists on disease outcome are not due to regulation of splenocyte function via the *β*-AR-Gs-cAMP pathway. Alternatively, the attenuating effects of *β*-AR agonists could reflect signaling via cAMP in other secondary lymphoid organs and/or at the affected joints.

Loss of *β*-agonist-induced cAMP response and *β*_2_-AR desensitization observed in this study is likely mediated, in part by, altered NE availability. While sympathetic activity is increased in RA (reviewed in [39]), no studies have directly assessed sympathetic nerve activity or NE turnover locally in lymphoid organs from animal models of RA; however, other data provide indirect support for this hypothesis. Loss and reorganization of sympathetic nerves in the spleen [48] are consistent with chronically high NE levels [40, 49] that subsequently auto-destroy NA nerves in target tissues [50]. Elevated plasma NE levels in AA rats at D1–5 or D10–21 after adjuvant challenge have been reported [49] and reflect spillover of NE released from sympathetic nerves in innervated tissues. Splenic and DLN NE concentrations in AA rats are reduced and elevated in chronic disease, respectively, supporting altered sympathetic nerve activity that is site specific [40]. Further, central and peripheral injection of proinflammatory cytokines, particularly, IL-1 and IL-6 (cytokines elevated in RA) increase SNS activity in the spleen [51–53]. 

Desensitization and downregulation of *β*-AR are dependent on p*β*_2_-AR by PKA and GRK, which occurs via site-specific phosphorylation at different serines/threonines [16–18]. Patterns of p*β*_2_-AR in splenocytes are disease specific. In AA rats, p*β*_2_-ARs at the PKA site are reduced on D21 and D28, while p*β*_2_-ARs at the GRK site are unchanged on D21 and elevated on D28 compared with each control group. p*β*_2_-AR by GRK2 uncouples *β*_2_-AR from Gs protein and facilitates the binding of *β*-arrestin to the receptor causing receptor downregulation by internalization [21]. Thus, the observed changes in splenocyte p*β*_2_-AR by PKA and GRK provide a mechanism for *β*_2_-AR downregulation and alternative second messenger signaling observed in splenocytes from AA rats.

Total *β*_2_-AR protein and its p*β*_2_-AR patterns in splenocytes and DLN cells are strikingly different in AA rats. On D21 and D28, the *β*_2_-AR_T_ protein levels in DLN cells are similar in all treatment groups, indicating no effect of AA induction on *β*_2_-AR expression. Unlike the spleen, p*β*_2_-AR_PKA_ are increased on D21 in DLN cells from CFA- and SMB-treated rats compared with the other control groups (MO and Saline). These findings suggest that the cell wall component of the adjuvant is responsible for increased p*β*_2_-AR_PKA_. By D28, p*β*_2_-AR_PKA_ site is reduced in CFA-, SMB-, and MO-treated rats compared with the Saline control group. In contrast, on D21 and D28, p*β*_2_-AR_GRK_ is greater in CFA- and SMB-treated rats than in rats treated with MO or Saline. These findings indicate a role for the mycobacterial cell wall component of CFA in driving p*β*_2_-AR_GRK_ and therefore implicate TLRs in regulating p*β*_2_-AR_GRK_, particularly TLR2 and TLR4 [54, 55]. Different GRKs (i.e., GRK2 versus GRK5/6) may be responsible for the disparate p*β*_2_-AR_GRK_ patterns in spleen and DLN cells [18, 27, 28]. p*β*_2_-AR by GRK5/6 shifts signaling into the ERK 1/2 cascade in HEK-293 cells treated with high physiological concentration of *β*-agonists [15, 20, 26]. 

Proinflammatory cytokines elevated in inflammatory arthritis may also contribute to the altered p*β*_2_-AR_GRK_, as IFN-*γ* and IL-6 or TNF-*α*, IL-1, and IL-12 induce different expression of GRKs and p*β*_2_-AR in PBMC from healthy controls [56–58]. Given that the SNS is crucial for maintaining homeostasis of the immune system, future research aimed at elucidating the mechanisms responsible for differential p*β*_2_-AR by PKA and GRKs and their consequences for immune function in AA is required to understand disease-specific SNS-immune crossregulation in RA. 

Of significance for RA, the ERK 1/2 signaling pathway increases IFN-*γ* production in AA CD4+ TH cells, cells that are prevalent in the arthritic joints and drive disease processes [59–61]. It is unclear whether stimulation of T cell *β*_2_-AR can increase IFN-*γ* by activating ERK 1/2 under conditions of chronically elevated NE release. If so, then it would provide a mechanism to explain the increased IFN-*γ* production after *β*_2_-AR stimulation in activated T cells [62], in splenic T cells shortly after onset of collagen-induced arthritis [7, 63] and DLN on day 28 after CFA-challenge in AA rats [41]. Many lymphocyte subsets are well known to express *β*_2_-AR, including CD4+TH1, CD8+ T cells, NK cells and B cells. Recently, CD4+FoxP3+ T regulatory (Treg) cells have been demonstrated to express *β*_2_-AR, and when stimulated, enhances their suppressive activity in a cAMP-PKA-dependent manner [64]. Further, the activation of *β*_2_-ARs on Treg cells enhances the ability of Treg cells to convert from CD4+CD62L+FoxP3+ T cells to FoxP3+ inducible Treg (iTreg) cells, an effect mediated by cAMP-PKA pathways. These cells are essential for maintaining tolerance and regulating adaptive immune responses [64, 65]. A lack of Treg cells is linked to the promotion of autoimmune disease development in both mice and humans [66–70]. Consistent with a role for the SNS in regulating Treg cell functions and their importance in autoimmune diseases, chemical sympathectomy with 6-hydroxydopamine increases the number of these cells in the spleen and lymph nodes in experimental autoimmune encephalomyelitis, a model for multiple sclerosis, an effect associated with reduced disease severity [71]. Cytokine profiles in AA rats indicate multiple lymphocyte subtypes are likely to contribute to the *β*_2_-AR changes observed in this study. Given that multiple T lymphocytes express *β*_2_-AR and the importance of interactions between various T cell subtypes, it will be necessary to determine the T cell subtypes responsible for the altered *β*_2_-AR expression, signaling and phosphorylation reported here. Future studies to understand the mechanism for altered *β*_2_-AR signaling in different lymphoid compartments in specific immune cell subtypes in our model and in RA are required. An understanding of *β*_2_-AR signaling changes that occur in RA also will be required before the potential to use *β*_2_-AR agonists are realized for treatment of RA. Understanding the mechanisms for altered *β*_2_-AR signaling may also lead to the improvement of current clinical use of *β*_2_-AR agonists.
